# HSPB8 is a Potential Prognostic Biomarker that Correlates With Immune Cell Infiltration in Bladder Cancer

**DOI:** 10.3389/fgene.2022.804858

**Published:** 2022-03-07

**Authors:** Zhiyong Tan, Shi Fu, Yinglong Huang, Xianzhong Duan, Yigang Zuo, Xiaorui Zhu, Haifeng Wang, Jiansong Wang

**Affiliations:** ^1^ Department of Urology, the Second Affiliated Hospital of Kunming Medical University, Yunnan Institute of Urology, Kunming, China; ^2^ Department of Urology, the Second People’s Hospital of Baoshan, Baoshan, China

**Keywords:** HSPB8, microarray, biomarker, bladder cancer, prognosis

## Abstract

**Background:** Heat shock protein B8 (HSPB8) is expressed in various cancers. However, the functional and clinicopathological significance of HSPB8 expression in bladder cancer (BC) remains unclear. The present study sought to elucidate the clinicopathological features and prognostic value of HSPB8 in BC.

**Methods:** A BC RNA-seq data set was obtained from The Cancer Genome Atlas Urothelial Bladder Carcinoma (TCGA-BLCA) database, and the external validation dataset GSE130598 was downloaded from the GEO database. Samples in the TCGA-BLCA were categorized into two groups based on HSPB8 expression. Differentially expressed genes (DEGs) between the two groups were defined as HSPB8 co-expressed genes. Gene set enrichment analysis (GSEA), protein-protein interaction networks, and mRNA-microRNA (miRNA) interaction networks were generated to predict the function and interactions of genes that are co-expressed with HSPB8. Finally, we examined immune cell infiltration and constructed a survival prediction model for BC patients.

**Results:** The expression level of HSBP8 has a significant difference between cancer samples and normal samples, and its diagnosis effect was validated by the ROC curve. 446 differential expressed genes between HSBP8 high-expression and HSBP8 low expression groups were identified. Gene enrichment analysis and GSEA analysis show that these differential gene functions are closely related to the occurrence and development of BC and the metabolic pathways of BC. The cancer-related pathways included Cytokine-cytokine receptor Interaction, Focal adhesion, and Proteoglycans in cancer. PPI and protein-coding gene-miRNA network visualized the landscape for these tightly bounded gene interactions. Immune cell infiltration shows that B cells, CD4+T cells, and CD8+T cells have strongly different infiltration levels between the HSBP8 high exp group and low exp group. The survival prediction model shows that HSBP8 has strong prognosis power in the BLCA cohort.

**Conclusion:** Identifying DEGs may enhance understanding of BC development’s causes and molecular mechanisms. HSPB8 may play an essential role in BC progression and prognosis and serve as a potential biomarker for BC treatment.

## Introduction

Bladder cancer (BC) occurs in the bladder mucosa and is the most common malignancy of the urinary system (Lenis et al., 2020). It is the fifth most common type of cancer worldwide, with an estimated 81,400 new cases and 17,000 related deaths in the United States in 2020 (Siegel et al., 2020). The most common histologic type of bladder cancer is uroepithelial cancer, accounting for more than 90% of all bladder cancers (Martinez Rodriguez., 2017). Based on the degree of muscle invasion, BC can be classified as non-muscle-invasive bladder cancer (NMIBC) and muscle-invasive bladder cancer (MIBC) (Kang et al., 2020). Recurrence remains a major challenge in the treatment of MIBC. Approximately 75% of patients initially present as NMIBC. Though these patients typically undergo aggressive treatments including, surgery, immunotherapy, chemotherapy, and radiotherapy, the patient response remains variable and unpredictable. About 10–30% of patients with NMIBC may relapse and progress to MIBC (Witjes et al., 2021), and the 5-years overall survival (OS) rate remains unsatisfactory. In addition, the cost of BC treatment poses a heavy burden on patients and society. The prognosis of BC patients is difficult to predict because there are no clinical biomarkers or parameters that can reliably reflect disease progression. Additionally, individual differences play important roles in determining the efficacy of thetreatment of BC. Therefore, clarifying the potential molecular mechanisms involved in BC carcinogenesis, proliferation, and recurrence and identifying novel potential biomarkers is crucial for early diagnosis, prognosis evaluation, and treatment.

Heat shock protein B8 (HSPB8, also known as small stress protein-like protein [sHSP22], protein kinase H11, E2-induced gene 1 protein [E2IG1], or alpha-crystallin C chain [CRYAC]), is a member of the small heat shock protein superfamily and contains a conserved α-crystallin domain at the C-terminal (F. Li et al., 2018). Various cellular functions have been linked to HSPB8, such as cytoskeleton stabilization, autophagy, oxidative stress, apoptosis, differentiation, and proliferation (L. L. Yu et al., 2021). In addition, studies have reported that HSPB8 exerts both beneficial and detrimental effects on cancer proliferation, invasion, and migration (Cristofani et al., 2021). For instance, HSPB8 expression is upregulated in breast cancer (Piccolella et al., 2017), multiple myeloma (Hamouda et al., 2014), lung cancer (L. L. Yu et al., 2021), and ovarian cancer (Suzuki et al., 2015). In these cancer types, HSPB8 promotes proliferation and suppresses apoptosis. For other tumors, including glioblastoma (Modem et al., 2011), prostate cancer (Hu et al., 2020), and hepatocarcinoma (Wang et al., 2020), HSPB8 is aberrantly methylated and expressed at low levels. Thus, the role of HSPB8 in cancer has attracted increasing attention. However, the expression and significance of HSPB8 in BC have not yet been characterized.

In this study, we investigated the correlation between HSPB8 expression and BC characteristics and analyzed the prognostic role of HSPB8 expression in BC based on RNA-seq data from TCGA and GEO datasets. In addition, we analyzed the expression levels of HSPB8 in BC and normal tissues and determined the correlation between HSPB8 expression and patient prognosis in terms of OS. Additionally, we examined the potential diagnostic and prognostic value of HSPB8 using patient data from the TGCA and GEO databases. Then, we elucidated its biological significance by performing enrichment analysis, molecular interaction network analysis, and immune infiltration correlation analysis. Our study highlights that HSPB8 may be a new predictor of BC diagnosis and prognosis and is a promising therapeutic target.

## Materials and Methods

### Data Download and Processing

We downloaded the gene expression data matrix of 433 samples (19 normal samples and 414 bladder cancer samples) from The Cancer Genome Atlas (TCGA) data portal (https://portal.gdc.cancer.gov/) as the training set. The microarray dataset GSE130598 analyzed using the GPL26612 platform was extracted from the Gene Expression Omnibus (GEO) database for the validation set. This dataset contains 24 normal samples and 24 tumor samples (Chandrashekar et al., 2020). The normalize between arrays function in the limma package (Ritchie et al., 2015) was used for background correction and data normalization.

### Differential Target Gene Co-expression Analysis

We grouped the target gene HSPB8 into high and low expression groups based on the median expression value in the test set derived from The Cancer Genome Atlas Urothelial Bladder Carcinoma (TCGA-BLCA) database. The differentially expressed genes (DEGs) between the high and low HSPB8 expression groups in the TCGA-BLCA and GSE130598 datasets were screened using the limma package. The DEG heatmap and volcano map was plotted using the ggplot2 package (Ito and Murphy, 2013). The intersection of the two groups of DEGs was examined using a Venn diagram. The correction of multiple testing was applied. All the DEGs had *p* < 0.05 and |log2FC|>1.

### Target Gene Correlation Analysis

We analyzed differences in HSPB8 expression between normal and tumor tissues. A ROC curve was generated to verify the diagnostic efficiency of HSPB8. The results indicate that HSPB8 is a significant diagnostic marker. The GEPIA platform (http://gepia.cancer-pku.cn/) (Tang et al., 2017) was used to analyze the body map of the median expression distribution of HSPB8 in tumors and normal samples. Additionally, patient base line data was shown (Table 1).

**TABLE 1:** TCGA-BLCA data set patient baseline data table.

Characteristic	Levels	Low expression of HSPB8	High expression of HSPB8	*p*
n		207	207	
T stage, n (%)	T1	5 (1.3%)	0 (0%)	0.013
	T2	60 (15.8%)	59 (15.5%)	
	T3	100 (26.3%)	96 (25.3%)	
	T4	21 (5.5%)	39 (10.3%)	
N stage, n (%)	N0	134 (36.2%)	105 (28.4%)	0.005
	N1	17 (4.6%)	29 (7.8%)	
	N2	28 (7.6%)	49 (13.2%)	
	N3	4 (1.1%)	4 (1.1%)	
M stage, n (%)	M0	110 (51.6%)	92 (43.2%)	1.000
	M1	6 (2.8%)	5 (2.3%)	
Pathologic stage, n (%)	Stage I	4 (1%)	0 (0%)	0.003
	Stage II	72 (17.5%)	58 (14.1%)	
	Stage III	77 (18.7%)	65 (15.8%)	
	Stage IV	53 (12.9%)	83 (20.1%)	
Gender, n (%)	Female	53 (12.8%)	56 (13.5%)	0.823
	Male	154 (37.2%)	151 (36.5%)	
Age, meidan (IQR)		69 (59, 75)	69 (61, 76.5)	0.280

### Friends/GO/KEGG Enrichment Analysis

To analyze the functional correlation between the key genes, we used the R package GOSemSim (G. Yu, 2020) to calculate the functional correlation of DEGs. The GO function annotation analysis is commonly used for large-scale gene enrichment studies and identified involved biological process (BP), molecular function (MF), and cellular component (CC) (Ashburner et al., 2000). KEGG is a widely used database that stores information about genomes, biological pathways, diseases, and drugs (Kanehisa et al., 2017). We used the clusterProfiler package (G. Yu et al., 2012) to perform the GO function analysis and KEGG pathway enrichment analysis on the DEGs related to BC.

### Gene Set Enrichment Analysis

Gene set enrichment analysis (GSEA) was used to evaluate the distribution of the genes in a pre-defined gene set. The genes were ranked by phenotype correlation to determine their contribution to the phenotype (Subramanian et al., 2007). We obtained c2. cp.v7.0. symbols.gmt from the MSigDB database and performed GSEA using the R package ClusterProfiler between the high and low HSPB8 expression groups in the TCGA-BLCA data set.

### Protein-Protein Interaction (PPI) Network Construction and Module Analysis

We constructed a molecular interaction network between high and low HSPB8 expression groups for DEGs. First, we performed protein-protein interaction (PPI) network analysis using the STRING database (Szklarczyk et al., 2019). Cytoscape is an open-source bioinformatics software program used to visualize molecular interaction networks. MCODE, a Cytoscape plug-in, was used to explore the PPI network hub genes. Then, hub genes with clear clustering were used as the target genes. MicroRNAs (miRNAs) that interact with the target genes were predicted using the TarBase (Karagkouni et al., 2018), miRecords (F. Xiao et al., 2009), and miRTarBase (H. Y. Huang et al., 2020) databases.

### Immune Cell Composition Assessment Analysis

Cell-type identification by estimating relative subsets of RNA transcripts (CIBERSORT) is based on the principle of linear support vector regression to deconvolve transcriptome expression matrices and estimate the composition and abundance of immune cells in a mixed cell population (B. Chen et al., 2018). We uploaded the gene expression matrix data to CIBERSORT and filtered the output for samples with *p* < 0.05 to derive the immune cell composition matrix. Heatmaps were drawn using the pheatmap package to demonstrate the composition distribution of the 22 immune cell types between the high and low HSPB8 expression groups. The core plot package was used to draw correlation heat maps to visualize the correlation of the composition of 22 immune cell types. ggplot2 was used to draw violin maps to visualize the differences in the composition of 22 immune cell types.

### Construction and Verification of Clinical Prediction Models

To assess whether HSPB8 expression and clinicopathological features can predict patient prognosis, we performed univariate and multivariate Cox regression analysis to understand whether the independent predictive power of risk scores and clinicopathological features are related to OS.

### Construction and Verification of Immune-Related Risk Prediction Model

We obtained immune-related genes from the ImmPort database (https://www.immport.org/) (Bhattacharya et al., 2018). This database assembles raw data from clinical trials, mechanistic studies, and cellular and molecular measurements. Templates for data representation and standard operating procedures were created to facilitate data transfer. We intersected the expressed genes from the high and low HSPB8 expression groups with immune-related genes. The TCGA-BLCA clinical data were also combined to construct risk prediction models for the immune-related genes. The least absolute shrinkage and selection operator (LASSO) algorithm was used to analyze the prognosis-related hub genes for dimensionality reduction analysis and feature selection (Tibshirani, 1996). The coefficients obtained by LASSO regression were weighted to each normalized gene expression value, and the following risk scoring formula was established:

We first examined the correlation between the expression of immune-related gene hub genes in different tumors in the TCGA database and the ability of the risk score to predict the prognosis of patients with different tumors. Subsequently, whether the risk scores combined with patient clinicopathological characteristics can predict OS was analyzed using univariate and multivariate Cox analysis, and a clinical prediction line graph (Nomogram) was constructed. Kaplan-Meier survival curves were generated to show survival differences. A log-rank test was performed to assess the difference in survival duration between the two patient groups. The correlation between clinical subgroup variables was also explored based on the risk scores.

### Statistical Analysis

All data were analyzed using R software (version 4.0.2). To compare continuous variables in two groups, normally distributed variables were analyzed using independent Student’s *t*-tests. Mann-Whitney U tests were used to analyze differences between non-normally distributed variables. The Chi-square test or Fisher’s exact test analyzed categorical data. The Wilcoxon test was used for two-group comparisons, and the Kruskal–Wallis test was used for multi-group comparisons. ROC curves and AUC values were obtained using the R package pROC. All statistical tests were two-tailed, and results with *p* < 0.05 were considered statistically significant.

## Results

### Differential Expression of Target Genes and Validation of Diagnostic Performance

**FIGURE 1:**
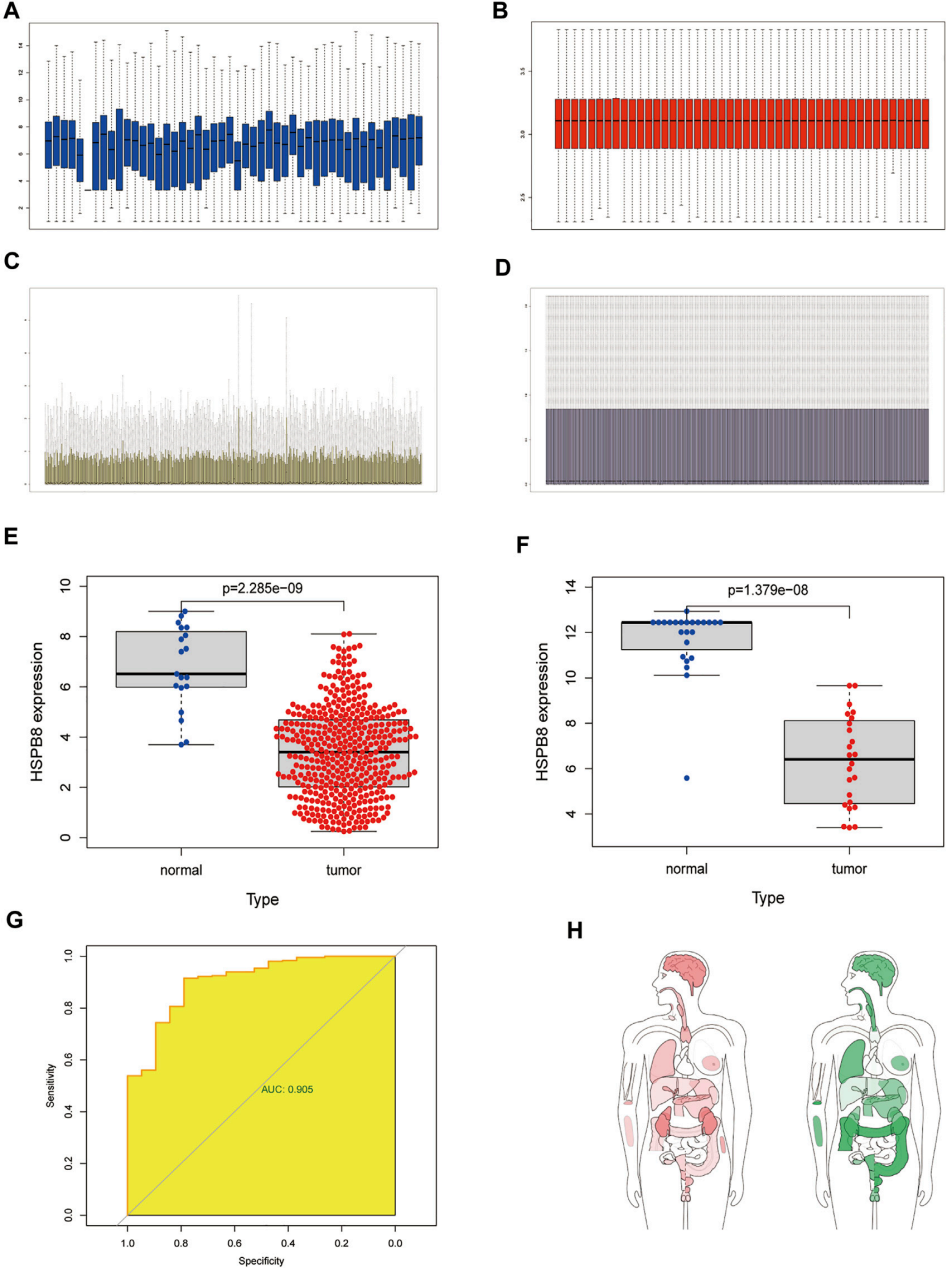
Differentially expressed target genes and diagnostic validation in bladder cancer. **(A)** mRNA expression profile from the GSE130598 dataset before normalization; **(B)** mRNA expression profile of the GSE130598 dataset after normalization; **(C)** mRNA expression profile from the TCGA-BLCA dataset before normalization; **(D)** mRNA expression profile of the TCGA-BLCA dataset after normalization; **(E)** Box plot of the differences in HSPB8 expression between the tumor and normal groups in theTCGA-BLCA dataset. Each point represents a sample. Blue represents normal tissue,and red represents tumor tissue; **(F)** Box plot of the differences in HSPB8 expression between the tumor and normal groups in the GSE130598 dataset. Each point represents a sample.Blue indicates normal tissue and red indicates tumor tissue. **(G)** Receiver operating characteristic(ROC) curve of HSPB8 in the TCGA-BLCA dataset; **(H)** HSPB8 distribution bodymap in tumor and normal samples, red represents tumor tissue (left), green represents normal tissue (right).

We performed background correction, and data normalization on the two data sets, and the gene expression before and after data normalization is shown in Figure 1 (A-B: GSE130598; C-D: TCGA-BLCA). We first compared the differences between high and low HSPB8 expression groups in the TCGA-BLCA and GSE130598 data sets (Figures 1E,F). A ROC curve was generated to verify the diagnostic efficiency of HSPB8 in the TCGA-BLCA dataset (AUC = 0.905). The results indicate that HSPB8 was a good predictive marker (Figure 1G). Next, the GEPIA platform was used to analyze the median expression distribution of HSPB8 in tumors and normal samples (Figure 1H). Additionally, patient base line data was shown (Table 1).

### Differential Expression of Target Gene

**FIGURE 2:**
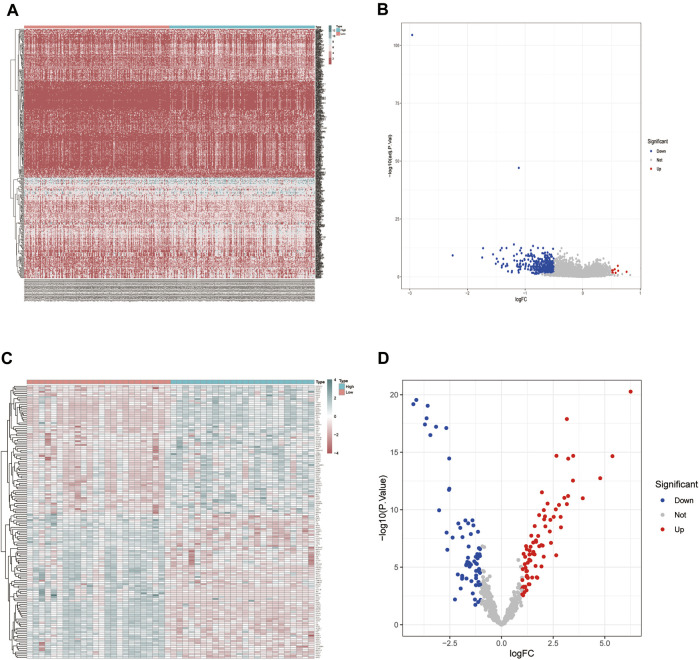
Differentially expressed gene distribution in the TCGA-BLCA dataset. **(A)** Heatmap of the HSPB8 expression between the high and low expression groups from the TCGA-BLCA dataset; **(B)** Volcano plot of gene expression in the high and low HSPB8 expression groups from the TCGA-BLCA dataset; **(C)** Heatmap of gene expression between the high and low HSPB8 expression groups in the GSE130598 dataset; **(D)** Volcano plot of gene expression between the high and low HSPB8 expression groups in the GSE130598 dataset.

We conducted differential expression analysis of the mRNA expression profile matrix derived from the TCGA-BLCA (Figures 2A,B) and GSE130598 datasets (Figures 2C,D). The differentially expressed genes between the high and low HSPB8 expression groups in the TCGA-BLCA and GSE130598 datasets are shown as heatmaps and volcano plots.

### Co-Expression Gene Friends Analysis and GO/KEGG Enrichment Analysis

**FIGURE 3:**
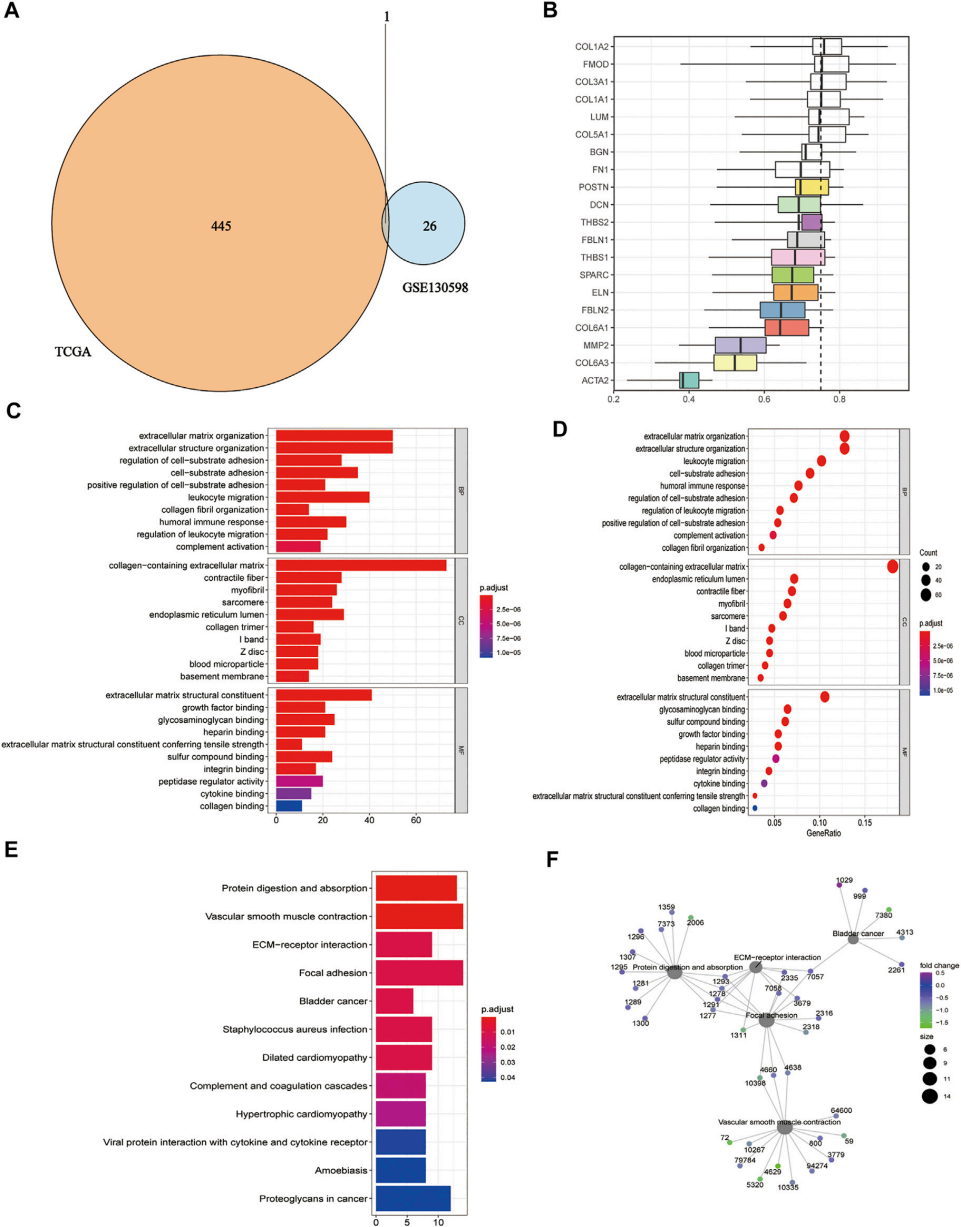
Gene ontology and KEGG enrichment analysis. **(A)** Venn diagram showing the co-expressed DEGs in the TCGA-BLCA and GSE130598 datasets; **(B)** Summary of functional similarities of the co-expressed genes; **(C)** GO enrichment analysis bar graph. The length of the bar represents the number of enriched genes, and the color represents the significance level (increasing from blue to red); **(D)** GO enrichment analysis bubble chart. The bubble size represents the number of enriched genes, and the color represents the significance level (increasing from blue to red); **(E)** KEGG enrichment analysis bar graph. The length of the bar represents the number of enriched genes, and the color represents the significance level (p.adjust < 0.05, increasing from blue to red, *p*-value adjusted for multiple comparisons); **(F)** KEGG enrichment analysis network diagram. Each point represents an enrichment term, and the color represents the significance level (*p* < 0.05, increasing from green to blue, *p*-values adjusted for multiple comparisons).

We took the intersection of DEGs between the high and low HSPB8 expression groups in the TCGA-BLCA (training set) and GSE130598 datasets (validation set) and displayed them as Venn diagrams (Figure 3A). HSPB8 was differentially expressed in both the training and validation sets. We compared the DEGs between the high and low HSPB8 expression groups using genes co-expressed with HSPB8. We identified co-expressed genes that are functionally related to HSPB8 by analyzing the functional correlation between genes co-expressed with HSPB8. The horizontal axis of the gene co-expression analysis reflects the correlation size, and the vertical axis shows the names of the co-expressed genes associated with the HSPB8 function (Figure 3B). GO, and KEGG enrichment analysis was performed using the clusterProfiler package. The GO enrichment analysis entries are presented as a bar chart (Figure 3C) and a bubble chart (Figure 3D). The KEGG enrichment analysis entries are shown as a bar graph (Figure 3E) and an enrichment graph (Figure 3F). Table 2 and Table 3 show the GO and KEGG enrichment entries that met the screening threshold, respectively. GO enrichment analysis showed that the genes co-expressed with HSPB8 were mostly associated with humoral immune response, complement activation, positive regulation of leukocyte migration, growth factor binding, peptidase regulatory activity, cytokine binding, and proteoglycan. The KEGG enrichment analysis showed that co-expressed genes were mostly enriched in cytokine-cytokine receptor interaction, protein digestion and absorption, proteoglycans in cancer, complement and coagulation cascade, viral protein-cytokine and cytokine receptor interaction, and bladder cancer and other related pathways. These results indicate that HSPB8 and its co-expressed genes may be related to BLCA development.

**TABLE 2:** The GO enrichment analysis of DEGs results between HSPB8 high expression group and low expression group. The pathways with p.adjust< 0.05 and qvalue< 0.05 are considered to be significantly enriched, showing the Top20 enrichment items of each category.

Ontology	ID	Description	adj.P	q
BP	GO:0030198	extracellular matrix organization	6.58E-23	5.24E-23
BP	GO:0043062	extracellular structure organization	6.58E-23	5.24E-23
BP	GO:0010810	regulation of cell-substrate adhesion	1.51E-11	1.20E-11
BP	GO:0031589	cell-substrate adhesion	2.59E-11	2.06E-11
BP	GO:0010811	positive regulation of cell-substrate adhesion	6.47E-11	5.15E-11
BP	GO:0050900	leukocyte migration	2.42E-10	1.92E-10
BP	GO:0030199	collagen fibril organization	2.11E-09	1.68E-09
BP	GO:0006959	humoral immune response	5.39E-08	4.29E-08
BP	GO:0002685	regulation of leukocyte migration	7.17E-08	5.71E-08
BP	GO:0006956	complement activation	2.02E-06	1.61E-06
BP	GO:0003012	muscle system process	2.12E-06	1.69E-06
BP	GO:0001503	ossification	2.12E-06	1.69E-06
BP	GO:0045785	positive regulation of cell adhesion	2.59E-06	2.06E-06
BP	GO:0006936	muscle contraction	3.98E-06	3.17E-06
BP	GO:0061448	connective tissue development	4.15E-06	3.31E-06
BP	GO:0051216	cartilage development	4.15E-06	3.31E-06
BP	GO:0001101	response to acid chemical	4.15E-06	3.31E-06
BP	GO:0060326	cell chemotaxis	6.16E-06	4.91E-06
BP	GO:0006958	complement activation, classical pathway	6.16E-06	4.91E-06
BP	GO:0002687	positive regulation of leukocyte migration	1.51E-05	1.20E-05
CC	GO:0062023	collagen-containing extracellular matrix	4.75E-45	3.83E-45
CC	GO:0043292	contractile fiber	1.05E-11	8.48E-12
CC	GO:0030016	Myofibril	1.16E-10	9.35E-11
CC	GO:0030017	Sarcomere	4.96E-10	4.00E-10
CC	GO:0005788	endoplasmic reticulum lumen	7.19E-10	5.79E-10
CC	GO:0005581	collagen trimer	1.35E-09	1.09E-09
CC	GO:0031674	I band	5.74E-09	4.63E-09
CC	GO:0030018	Z disc	1.01E-08	8.14E-09
CC	GO:0072562	blood microparticle	5.36E-08	4.32E-08
CC	GO:0005604	basement membrane	2.98E-07	2.40E-07
CC	GO:0034774	secretory granule lumen	1.81E-06	1.46E-06
CC	GO:0060205	cytoplasmic vesicle lumen	2.09E-06	1.68E-06
CC	GO:0031983	vesicle lumen	2.17E-06	1.75E-06
CC	GO:0031093	platelet alpha granule lumen	2.61E-05	2.11E-05
CC	GO:0005796	Golgi lumen	2.85E-05	2.30E-05
CC	GO:0005583	fibrillar collagen trimer	3.07E-05	2.47E-05
CC	GO:0098643	banded collagen fibril	3.07E-05	2.47E-05
CC	GO:0098644	complex of collagen trimers	3.07E-05	2.47E-05
CC	GO:0031091	platelet alpha granule	4.79E-05	3.86E-05
CC	GO:0032432	actin filament bundle	5.29E-05	4.26E-05
MF	GO:0005201	extracellular matrix structural constituent	3.31E-29	2.82E-29
MF	GO:0019838	growth factor binding	6.23E-10	5.32E-10
MF	GO:0005539	glycosaminoglycan binding	7.45E-09	6.36E-09
MF	GO:0008201	heparin binding	1.86E-08	1.58E-08
MF	GO:0030020	extracellular matrix structural constituent conferring tensile strength	8.94E-08	7.62E-08
MF	GO:1901681	sulfur compound binding	1.30E-07	1.10E-07
MF	GO:0005178	integrin binding	3.55E-07	3.03E-07
MF	GO:0061134	Peptidase regulator activity	5.31E-06	4.53E-06
MF	GO:0019955	cytokine binding	8.02E-06	6.84E-06
MF	GO:0005518	collagen binding	1.09E-05	9.30E-06
MF	GO:0030021	extracellular matrix structural constituent conferring compression resistance	1.41E-05	1.20E-05
MF	GO:0050840	extracellular matrix binding	1.72E-05	1.46E-05
MF	GO:0050839	cell adhesion molecule binding	2.60E-05	2.22E-05
MF	GO:0043394	proteoglycan binding	3.28E-05	2.79E-05
MF	GO:0001968	fibronectin binding	4.88E-05	4.17E-05
MF	GO:0048407	platelet-derived growth factor binding	6.68E-05	5.70E-05
MF	GO:0003823	antigen binding	7.62E-05	6.50E-05
MF	GO:0008307	structural constituent of muscle	0.000181	0.000155
MF	GO:0004857	enzyme inhibitor activity	0.000268	0.000228
MF	GO:0030414	Peptidase inhibitor activity	0.000299	0.000255

**TABLE 3:** KEGG pathway enrichment analysis of DEGs between HSPB8 high expression and low expression groups.

ID	Description	p.adjust	Qvalue	Count
hsa04060	Cytokine-cytokine receptor interaction	0.045336	0.041118	15
hsa04270	Vascular smooth muscle contraction	0.000189	0.000171	14
hsa04510	Focal adhesion	0.009932	0.009008	14
hsa04974	Protein digestion and absorption	0.000111	0.000101	13
hsa05205	Proteoglycans in cancer	0.042693	0.038721	12
hsa04512	ECM-receptor interaction	0.009932	0.009008	9
hsa05150	*Staphylococcus aureus* infection	0.009932	0.009008	9
hsa05414	Dilated cardiomyopathy	0.009932	0.009008	9
hsa04610	Complement and coagulation cascades	0.018281	0.01658	8
hsa05410	Hypertrophic cardiomyopathy	0.023775	0.021563	8
hsa04061	Viral protein interaction with cytokine and cytokine receptor	0.042398	0.038453	8
hsa05146	Amoebiasis	0.042693	0.038721	8
hsa05219	Bladder cancer	0.009932	0.009008	6
hsa00590	Arachidonic acid metabolism	0.042693	0.038721	6

### KEGG Enrichment Analysis Pathway Diagram

**FIGURE 4:**
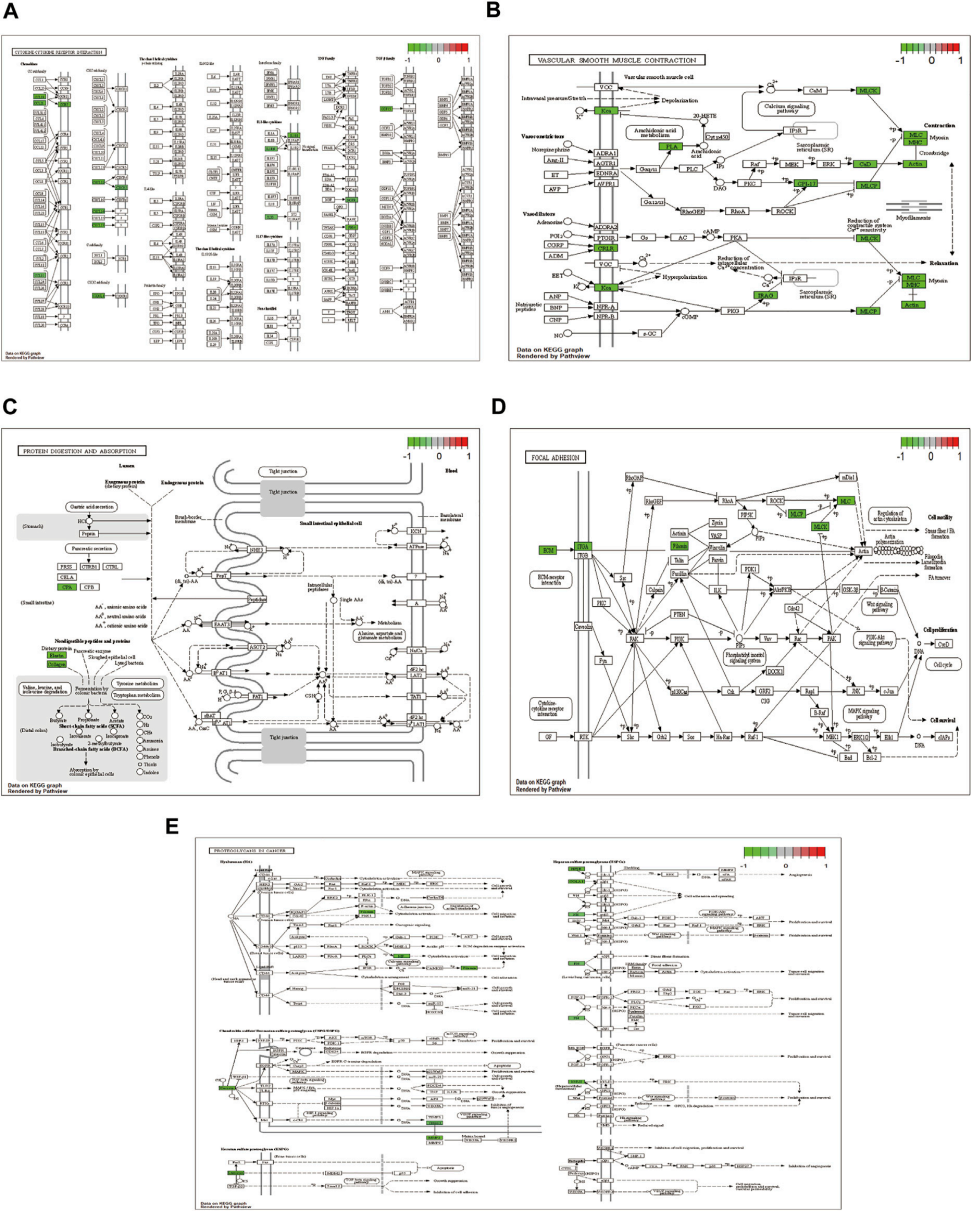
KEGG enrichment analysis showing **(A)** cytokine-cytokine receptor interaction; **(B)** vascular smooth muscle contraction; **(C)** focal adhesion; **(D)** protein digestion and absorption; and **(E)** proteoglycans in cancer pathway diagram.

The pathways with >10 genes enriched by KEGG enrichment analysis included cytokine-cytokine receptor interactions, vascular smooth muscle contraction, adhesion, protein digestion, absorption, and proteoglycan pathways in cancer (Figure 4). Genes labeled in green in the pathway map reflect DEGs.

### Protein-Protein Interaction Network Construction

**FIGURE 5:**
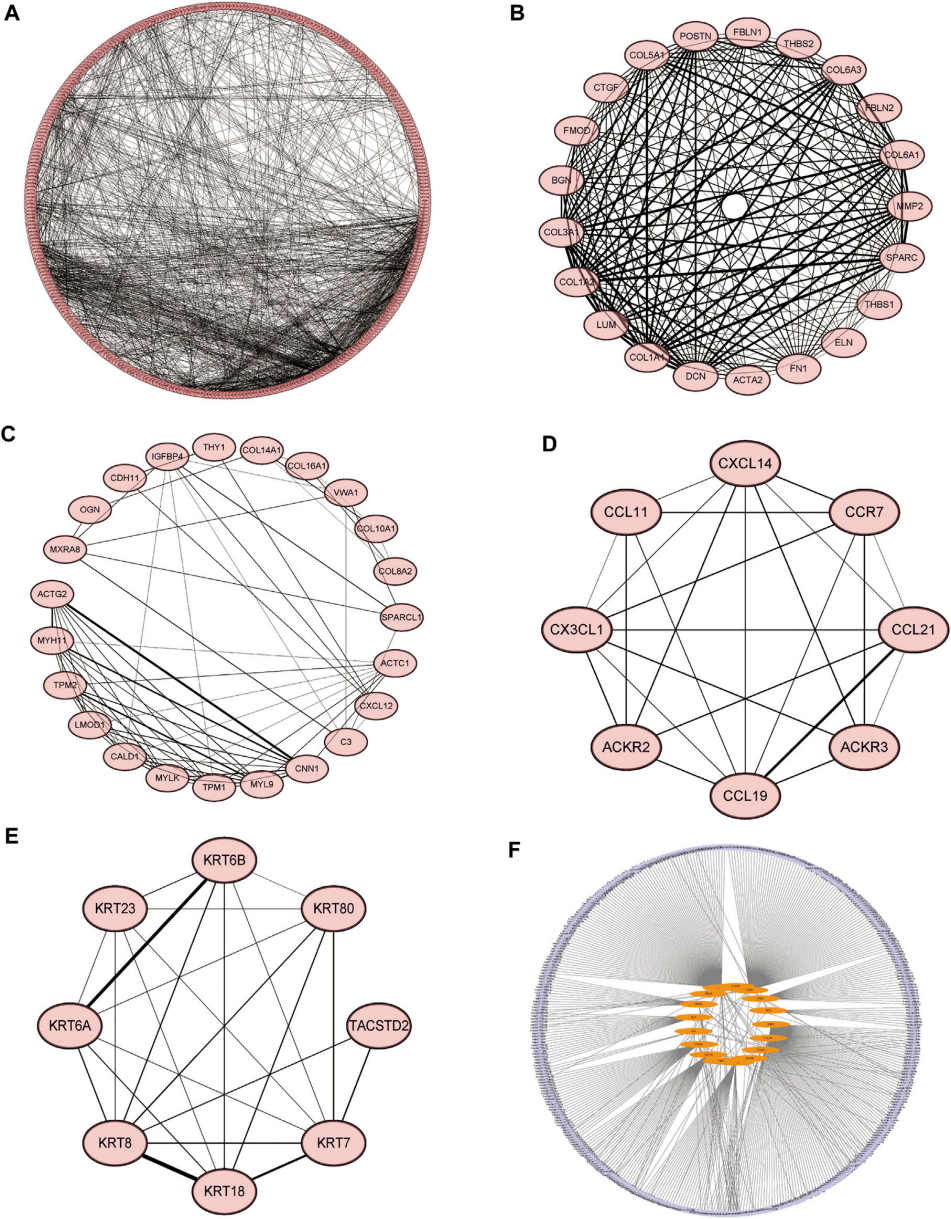
Molecular interaction networks. **(A)** Protein-protein interaction network analysis was performed on DEGs between the high and low HSPB8 expression groups using the STRING database. Cytoscape was used for visualization. **(B–E)** The MCODE plug-in was used to analyze hub genes, which include the four groups with the largest number of clusters; **(F)** The most closely linked hub genes were used as target genes, and the miRNAs that interact with the target genes were predicted by the TarBase, miRecords, and miRTarBase databases to construct a molecular interaction network.

We constructed molecular interaction networks between the DEGs’ high and low HSPB8 expression groups. PPI network analysis was performed using the STRING database and visualized using Cytoscape (Figure 5A). Hub genes were further analyzed using the MCODE plug-in (Figures 5B–E). We used the most closely linked set of hub genes obtained from the MCODE analysis as target genes and predicted the miRNAs that interact with the target genes using the TarBase, miRecords, and miRTarBase databases (Figure 5F).

### Gene Set Enrichment Analysis

**FIGURE 6:**
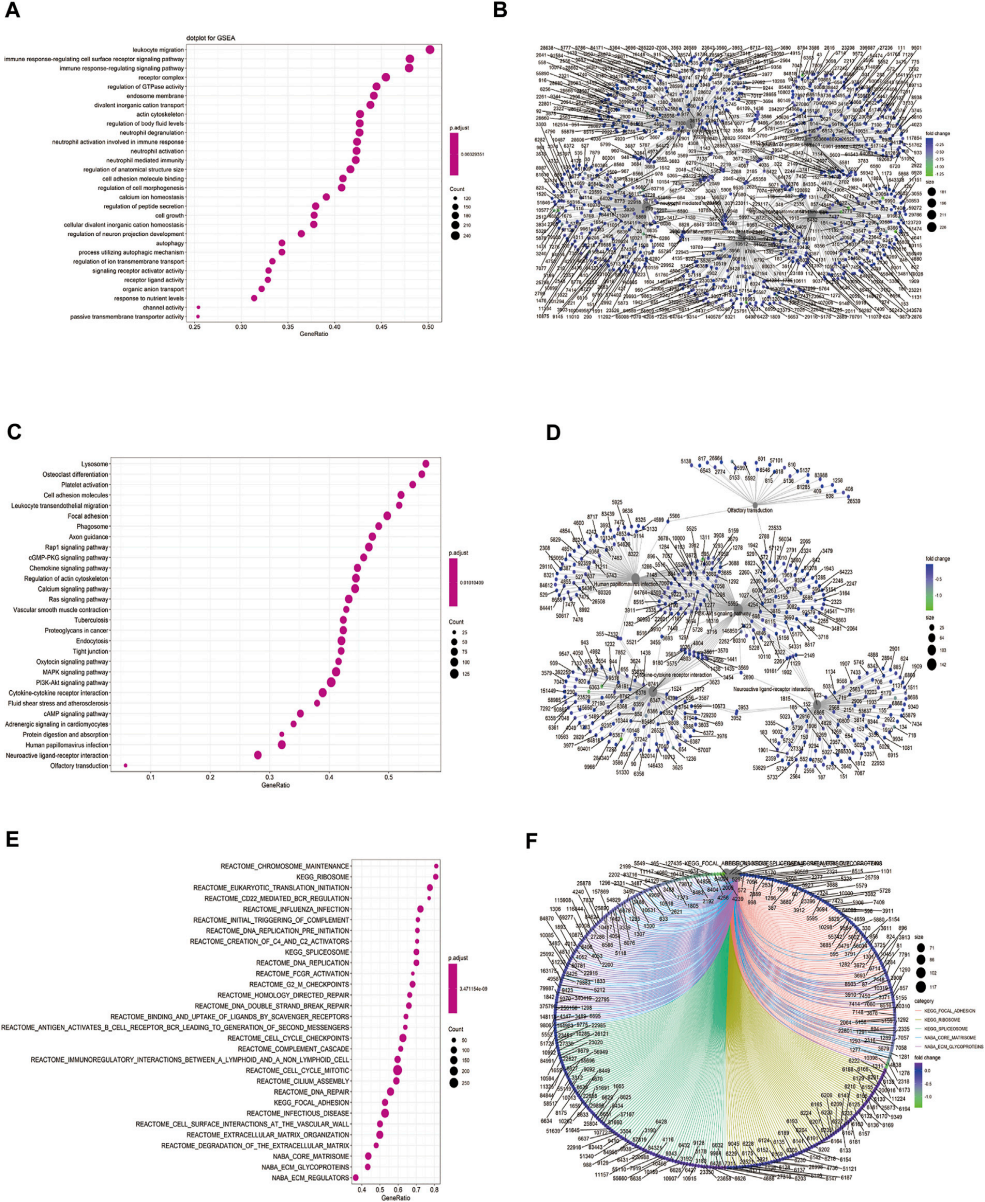
Gene set enrichment analysis (GSEA). **(A)** Bubble chart showing the GO terms enriched between the high and low HSPB8 expression groups in the TCGA-BLCA dataset; **(B)** Enrichment plots of the GO terms between the high and low HSPB8 expression groups in the TCGA-BLCA dataset; **(C)** Bubble chart of the enriched KEGG terms between the high and low HSPB8 expression groups in the TCGA-BLCA data set; **(D)** Enrichment plot of the KEGG terms between the high and low HSPB8 expression groups in the TCGA-BLCA dataset; **(E)** Bubble chart of the enriched GSEA entries between the high and low HSPB8 expression groups in the TCGA-BLCA dataset; **(F)** Chordogram of GSEA term enrichment between the high and low HSPB8 expression groups in the TCGA-BLCA dataset.

We performed GSEA on the TCGA-BLCA dataset using the clusterProfiler package to analyze the gene expression matrix. The file, c2. all.v7.0. entrez.gmt, was used as the reference gene set. The enrichment analysis was performed using the gseGO and gseKEGG functions. The top 30 entries with p. adjust <0.05 were visualized (Figures 6A–F; Table 4). The results were mostly enriched in the response group signaling pathways in cancer, NF-kB pathway, lymphocyte pathway, CD40 pathway, IL17, IL3, IL5, P53, ERK5, NO2IL12, ALK2 pathway, and cytokine linkage pathway.

**TABLE 4:** TCGA-BLCA data set HSPB8 high expression group and low expression group Top30 NES absolute value GSEA enrichment analysis results list.

ID	Enrichment score	NES	p.adjust	Qvalues
BIOCARTA_STEM_PATHWAY	−0.9295	−2.5952	3.30E-09	1.25E-09
BIOCARTA_IL17_PATHWAY	−0.9157	−2.5566	3.30E-09	1.25E-09
BIOCARTA_CLASSIC_PATHWAY	−0.9850	−2.4411	3.30E-09	1.25E-09
BIOCARTA_LONGEVITY_PATHWAY	−0.8684	−2.4246	3.30E-09	1.25E-09
BIOCARTA_IL3_PATHWAY	−0.8655	−2.4164	3.30E-09	1.25E-09
BIOCARTA_ERYTH_PATHWAY	−0.8596	−2.3998	3.30E-09	1.25E-09
REACTOME_ANCHORING_FIBRIL_FORMATION	−0.8584	−2.3965	3.30E-09	1.25E-09
BIOCARTA_GRANULOCYTES_PATHWAY	−0.8576	−2.3943	3.30E-09	1.25E-09
SA_MMP_CYTOKINE_CONNECTION	−0.8574	−2.3937	3.30E-09	1.25E-09
BIOCARTA_NO2IL12_PATHWAY	−0.8546	−2.3859	3.96E-09	1.50E-09
REACTOME_SEROTONIN_RECEPTORS	−0.9333	−2.3830	3.30E-09	1.25E-09
REACTOME_ENDOSOMAL_VACUOLAR_PATHWAY	−0.9206	−2.3566	3.77E-09	1.42E-09
PID_ALK2_PATHWAY	−0.9189	−2.3523	4.17E-09	1.57E-09
REACTOME_WNT5A_DEPENDENT_INTERNALIZATION_OF_FZD4	−0.8415	−2.3494	7.93E-09	2.99E-09
BIOCARTA_ASBCELL_PATHWAY	−0.9296	−2.3380	3.30E-09	1.25E-09
BIOCARTA_RELA_PATHWAY	−0.8327	−2.3249	1.29E-08	4.87E-09
BIOCARTA_CD40_PATHWAY	−0.8282	−2.3121	1.71E-08	6.45E-09
REACTOME_TYPE_I_HEMIDESMOSOME_ASSEMBLY	−0.8964	−2.2947	3.51E-08	1.33E-08
BIOCARTA_FIBRINOLYSIS_PATHWAY	−0.8964	−2.2889	5.58E-09	2.11E-09
BIOCARTA_AGPCR_PATHWAY	−0.8914	−2.2818	5.16E-08	1.95E-08
BIOCARTA_D4GDI_PATHWAY	−0.8920	−2.2777	6.62E-09	2.50E-09
REACTOME_REGULATION_OF_GLYCOLYSIS_BY_FRUCTOSE_2_6_BISPHOSPHATE_METABOLISM	−0.8917	−2.2768	6.62E-09	2.50E-09
REACTOME_CREB1_PHOSPHORYLATION_THROUGH_THE_ACTIVATION_OF_ADENYLATE_CYCLASE	−0.8905	−2.2737	7.02E-09	2.65E-09
PID_ARF6_DOWNSTREAM_PATHWAY	−0.8128	−2.2693	6.79E-08	2.57E-08
BIOCARTA_BLYMPHOCYTE_PATHWAY	−0.8867	−2.2642	8.53E-09	3.22E-09
BIOCARTA_CTL_PATHWAY	−0.8920	−2.2437	3.30E-09	1.25E-09
BIOCARTA_IL5_PATHWAY	−0.8751	−2.2401	1.84E-07	6.94E-08
BIOCARTA_MONOCYTE_PATHWAY	−0.8741	−2.2376	1.98E-07	7.49E-08
BIOCARTA_P53_PATHWAY	−0.8569	−2.2245	3.30E-09	1.25E-09
BIOCARTA_EPONFKB_PATHWAY	−0.8684	−2.2229	2.59E-07	9.77E-08

### Differential Analysis of Immune Cell Composition

**FIGURE 7:**
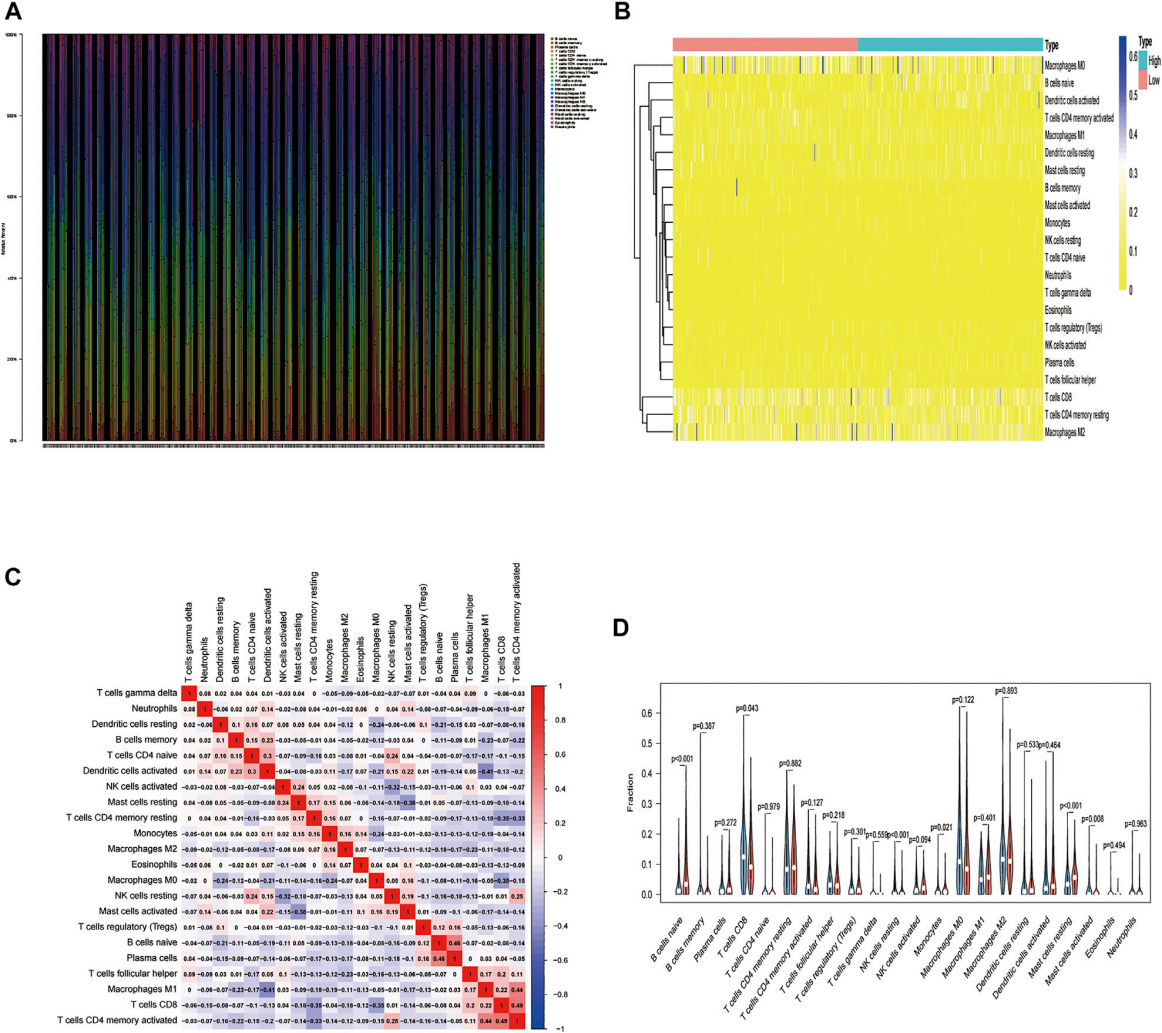
The relationship between HSPB8 expression and immune cell composition in the TCGA-BLCA data set. **(A)** Immune cell composition in the high and low HSPB8 expression groups. The proportion of composition by 22 immune cell types in tumor samples is shown in the stacked histogram; **(B)** The relationship between immune cell composition and HSPB8 expression; **(C)** The correlation of immune cell composition in the 22 samples. Red represents positive correlations, and blue represents negative correlations; **(D)** Immune cell composition in the high and low HSPB8 expression groups, as analyzed using the CIBERSORT algorithm.

**FIGURE 8:**
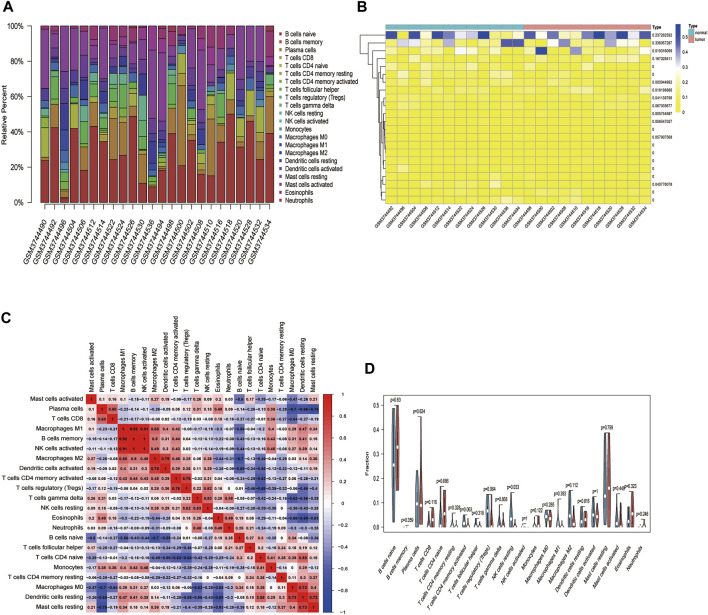
The relationship between HSPB8 expression and immune cell composition in the GSE130598 data set. **(A)** Immune cell composition in the high and low HSPB8 expression groups. The proportion of composition by 22 immune cell types in tumor samples is shown in the stacked histogram; **(B)** The relationship between immune cell composition and HSPB8 expression; **(C)** The correlation of immune cell composition in the 22 samples. Red represents positive correlations, and blue represents negative correlations; **(D)** Immune cell composition in the high and low HSPB8 expression groups, as analyzed using the CIBERSORT algorithm.

We used the CIBERSORT algorithm to deconvolute the gene expression matrices and derive the immune cell composition matrix (Figure 7, TCGA-BLCA dataset; Figure 8, GSE130598 dataset). The par function calculated the immune cell percentage, and stacked histograms (Figure 7A, Figure 8A) and heatmaps (Figure 7B, Figure 8B) were plotted. A correlation heat map was plotted to visualize the correlation of composition by 22 immune cell types (Figure 7C, Figure 8C), with red representing positive correlations and blue representing negative correlations. The differences in the composition by the 22 immune cell types amounts were plotted in violin plots (Figure 7D, Figure 8D). The results showed significant differences between the groups in the composition of various immune cells, including B cells, CD4^+^ T cells, and CD8^+^ T cells.

### Construction of a Prognostic Risk Model of Immune-Related Co-expressed Genes of HSPB8

**FIGURE 9:**
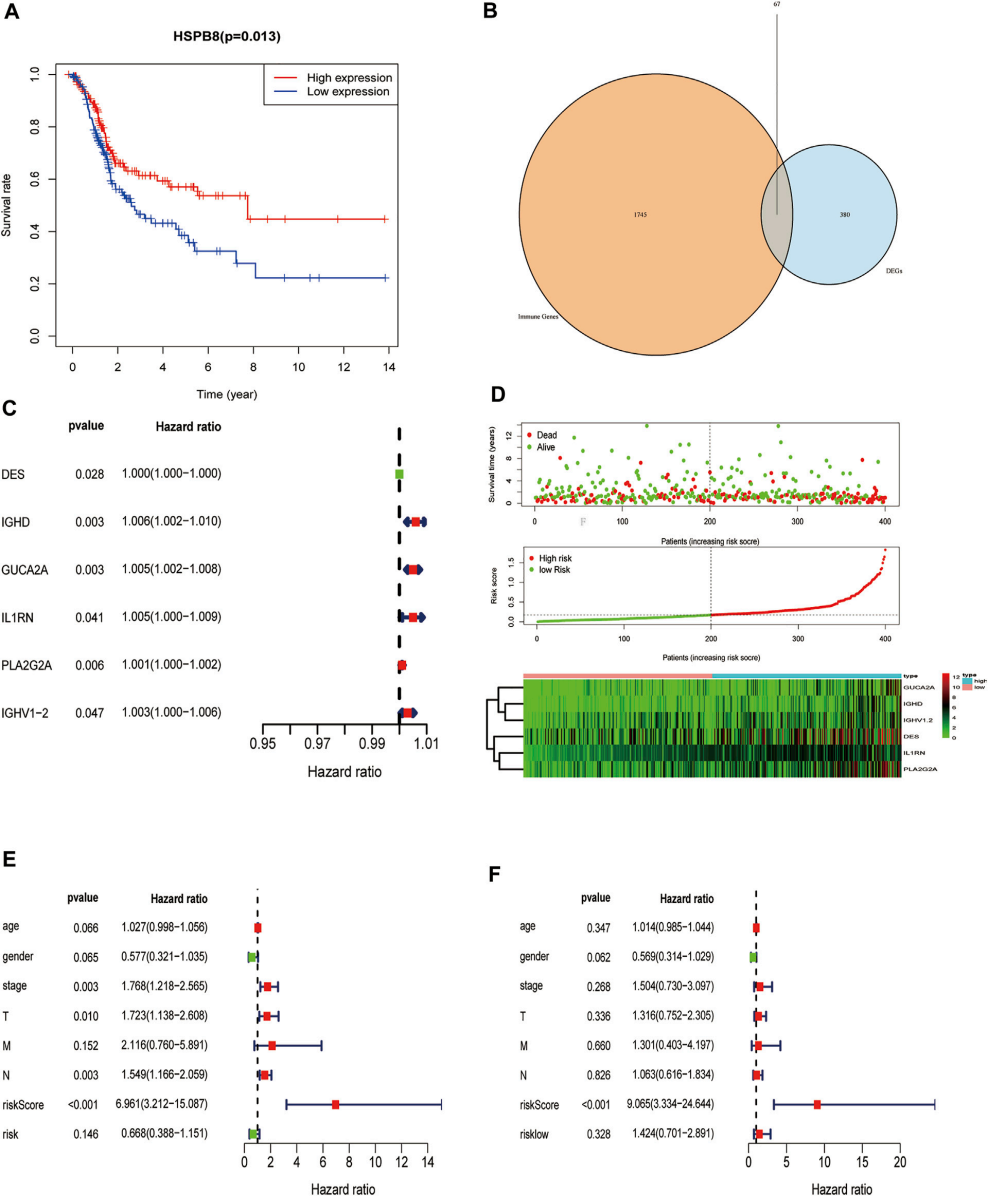
Construction of a prognostic risk model of immune-related genes co-expressed with HSPB8. **(A)** HSPB8 expression affects the overall survival of BC patients; **(B)** The intersection of HSPB8 co-expressed genes with immune-related genes; **(C)** Combination of TCGA-BLCA clinical data, HSPB8 expression, and co-expressed immune-related genes were used to construct the risk prediction model; **(D)** The risk curve of HSPB8 co-expressed, immune-related gene risk model (top), survival status (middle), and risk heatmap (bottom); **(E)** Forest plot showing univariate Cox regression analysis of the predictive power of risk scores combined with clinicopathological characteristics of patients for BC prognosis; **(F)** Forest diagram showing multivariate Cox analysis of the predictive ability of risk scores combined with clinicopathological characteristics of patients for BC prognosis.

We examined whether HSPB8 expression affects the overall survival of BLCA patients (Figure 9A). We first intersected the genes co-expressed with HSPB8 and immune-related genes (Figure 9B). Next, we combined clinical data from the TCGA-BLCA dataset to construct a risk prediction model for this set of immune-related genes (Figure 9C) and plotted the risk curves (top), survival status (middle), and risk heat map (bottom) (Figure 9D). To demonstrate the personalized assessment of patient prognosis using risk scores combined with clinicopathological features, we tested the immune-related gene expression risk models in different tumors from the TCGA database and the predictive power of risk scores to determine patients’ prognosis with different patients tumors. Then, the predictive ability of risk scores combined with clinicopathological features to project BLCA patient prognosis was analyzed using univariate and multifactorial Cox analysis (Figures 9E,F; Table 5).

**TABLE 5:** Univariate and multifactorial Cox regression analysis.

Characteristics	Total(N)	Univariate analysis			Multivariate analysis	
		HR (95% CI)	*p* Value		HR (95% CI)	*p* Value
T	379					
T1 & T2	124					
T3 & T4	256	2.199 (1.515–3.193)	< 0.001		3.064 (0.904–10.391)	0.072
N	369					
N0	239					
N1 & N2 & N3	131	2.289 (1.678–3.122)	< 0.001		2.005 (1.157–3.472)	0.013
M	213					
M0	202					
M1	11	3.136 (1.503–6.544)	0.002		1.204 (0.461–3.145)	0.705
Stage	411					
Stage I & Stage II	134					
Stage III & Stage IV	278	2.310 (1.596–3.342)	< 0.001		0.567 (0.152–2.123)	0.4
Gender	413					
Female	109					
Male	305	0.849 (0.616–1.169)	0.316			
Age	413					
≤70	234					
>70	180	1.421 (1.063–1.901)	0.018		1.197 (0.748–1.915)	0.453
HSPB8	413	1.082 (1.006–1.163)	0.033		1.049 (0.930–1.184)	0.438

### Decreased Expression of HSPB8 Predicts a Poor Prognosis in Patients With BLCA

**FIGURE 10:**
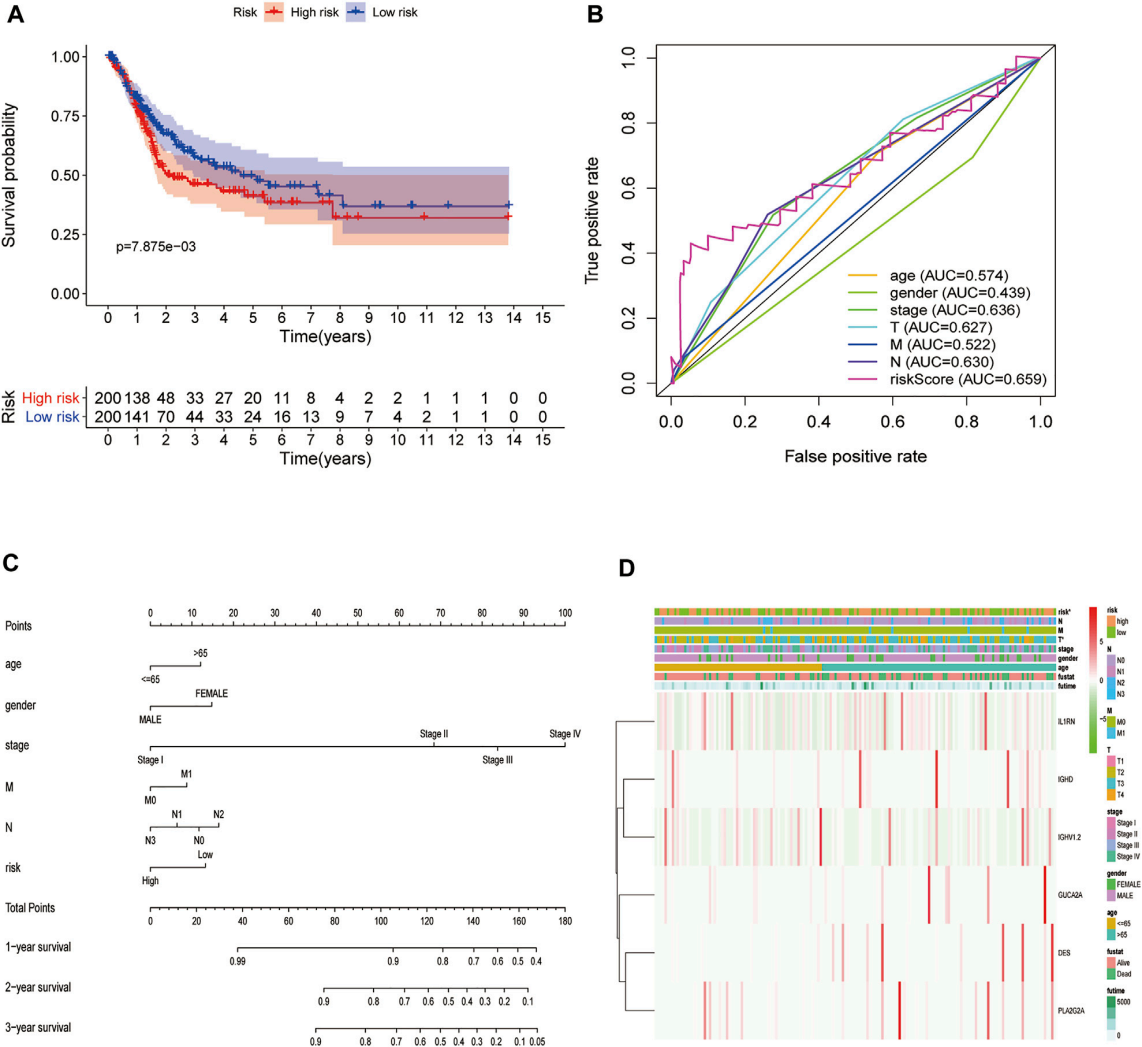
Decreased expression of HSPB8 predicts a poor prognosis in patients with BLCA. **(A)** Overall survival between the high and low-risk groups from the TCGA-BLCA database; **(B)** The BLCA prognostic value of each clinical variable and its accuracy ROC curve; **(C)** Construction of a clinical prediction nomogram; **(D)** Correlation analysis of clinical subgroup variables based on the risk score and the differences between the clinical subgroups shown as a heatmap.

To further explore the impact of the risk model gene set on the BLCA patient survival and prognosis, we analyzed the OS between high and low-risk groups using it as a risk factor in the clinical survival data from the TCGA database (Figure 10A). The survROC package was used to predict whether the clinical variables included in the analysis accurately predicted BLCA patient survival and prognosis based on the AUC value of the prognostic risk score (AUC = 0.659). The results indicated that the gene set had some accuracy for the OS prognosis of BLCA. A nomogram for clinical prediction was also constructed (Figure 10C). Correlation analysis of the clinical subgroup variables was explored based on risk scores to demonstrate differences between clinical subgroups (Figure 10D).

## Discussion

BC is among the most prevalent malignancies of the genitourinary system and has been identified as the fourth and 10th leading cause of cancer-related deaths in males and females, respectively (Ma et al., 2018). The pathogenesis of BC is complex and involves several factors, including intrinsic genetic factors and extrinsic environmental factors, such as smoking, chemical pollution, genetic mutation, and single nucleotide polymorphisms (Cai et al., 2019; Turner et al., 2019; Xu Chenyang et al., 2020). With the rapid advancements in molecular biology techniques, BC research and treatment have been greatly improved. However, the long-term prognosis of BC remains unsatisfactory, and the pathogenesis associated with BC progression remains elusive. Microarray and bioinformatics analyses have significantly enhanced our understanding of disease occurrence and the development of molecular mechanisms. These analyses are necessary to explore genetic alternations and identify potential diagnostic biomarkers. However, when using single microarray datasets, high false-positive rates and biased results have been observed. Therefore, novel, highly specific, sensitive, and effective biomarkers are urgently required for BC diagnosis and treatment. This study explored the gene expression profile and BC pathogenesis using high-throughput transcriptome data obtained from TCGA and GEO datasets. We show that the expression of HSPB8, which is involved in the regulation of cell proliferation, apoptosis, and carcinogenesis, is related to BC progression and prognosis. We comprehensively analyzed HSPB8 expression its clinical relevance and explored its potential diagnostic and prognostic value in BC.

Analysis of hub genes using the MCODE plug-in identified CCL21 as a gene related to HSPB8 expression in BC. Recent studies have shown that chemokines such as IL-8 can enhance chemoresistance and cancer stem cell-like properties (Lu et al., 2016). The atypical ACKR4, which is expressed in epithelial cells of the bladder, is a high-affinity receptor for CCL21. Further, CCL21 perfusion in the rat bladder increases bladder excitability and increases c-fos activity in spinal cord neurons (Offiah et al., 2016). CXCL14 (also known as BRAK) is involved in tumorigenesis. Pancreatic and prostate cancers show increased CXCL14 expression, while other cancer types, including cancers of breast, kidneys, and cervix, show downregulated CXCL14 expression (Nagarsheth et al., 2017). Furthermore, CXCL14 expression is inhibited by DNA methylation in lung cancer cells, resulting in reduced tumor growth.

GO enrichment analysis of genes co-expressed with HSPB8 were enriched in humoral immune response, complement activation, positive regulation of leukocyte migration, growth factor binding, peptidase regulatory activity, cytokine binding, proteoglycan binding, and peptidase inhibitor activity pathways. Complement system activation is tightly regulated by a network of proteins known as the complement activation regulator, limiting host complement activation and thus preventing self-injury (Amet et al., 2012). Excessive complement activation in the tumor microenvironment is associated with inflammation and tumor growth (Reis et al., 2019). Non-covalent interaction of proteoglycans with HyA (an important non-proteoglycan) occurs via hyaluronan binding motifs (Shih and Varghese, 2019). Tumor cells express different membrane proteins such as endothelial growth factor receptor (EGFR) and cell surface proteoglycans, making it possible for molecules to bind specifically to these proteins (Y. F. Xiao et al., 2015). KEGG enrichment analysis showed gene enrichment in cytokine-cytokine receptor interactions, vascular smooth muscle contraction, adhesion, protein digestion and absorption, cancer smooth muscle contraction, and proteoglycan pathway in cancer. Cytokine-cytokine receptor activation leads to key immune signaling pathways that regulate cancer development and progression (X. Chen et al., 2020). Furthermore, apart from differentially expressed genes in BC, miRNA expression affects cell cycle progression, epithelial-mesenchymal transition, cytokine-cytokine receptor interactions, and downstream cancer pathways, including phosphatidylinositol 3-kinase (PI3K)-Akt signaling and mitogen-activated protein kinase signaling pathways (Lee et al., 2018).

In the present study, GSEA was performed to investigate the potential signaling pathways in BC with high HSPB8 expression. Our results suggested that BC patients with high HSPB8 expression have increased gene expression related to the response group signaling pathway, NF-κB pathway, lymphocyte pathway, CD40 pathway, IL17, IL3, IL5, P53, ERK5, NO2IL12, ALK2 pathway, and cytokine linkage pathway in cancer. Recent studies have shown that inflammatory signaling pathways are involved in carcinogenesis via activation of NF-κB signaling (Sun et al., 2019), which may act as a downstream pathway regulating BC proliferation and progression (Xu Jing et al., 2020). Immune response pathways are involved in inflammatory bowel disease, leukocyte transendothelial migration, complement system, coagulation cascade, chemokine signaling pathways, toll-like receptor signaling pathways, B-cell receptor signaling pathway, systemic lupus erythematosus, platelet activation, and IL-17 signaling pathway (Du et al., 2019).

The tumor microenvironment affects the occurrence and recurrence of tumors and plays an important role in tumor immunotherapy outcomes. Tumor-infiltrating immune cells are an indispensable component of the tumor microenvironment, and their composition and distribution are related to cancer prognosis (B. Li et al., 2016). Previous studies have reported that the location, type, and density of inflammatory infiltrating cells in colorectal cancer are better predictors of survival than clinical and histopathological factors (Galon et al., 2006). Given the critical role of the tumor microenvironment in cancer progression, and because tumor-infiltrating immune cells are an integral part of the tumor microenvironment, we investigated the relationship between HSPB8 expression status and immune infiltration in BC. Our results showed significant differences between groups for various immune cells such as B cells, CD4^+^ T cells, and CD8^+^ T cells. Huang et al. (Y. Huang et al., 2015)observed that CD8^+^ T cells are key factors influencing tumor immunotherapy. Their role depends on the composition of the accompanying inflammatory cells, including macrophages, B cells, and CD4 TILs.

Similarly, Matsumoto et al. (Matsumoto et al., 2016) found that increased CD4^+^ and CD8^+^ T cell infiltration is associated with better clinical outcomes in triple-negative breast cancer. In BC, CD8^+^ T cells and memory-activated CD4^+^ T cells showed increased infiltration and abundance in a high-TMB group that correlated with prolonged OS and a lower risk of recurrence (Zhang et al., 2020). Therefore, we inferred that HSPB8 impacts the immune microenvironment of BC participates in the regulation of BC tumor immunity can be used as a prognostic indicator for BC and can reflect the immune status of patients.

Despite performing a thorough computational analysis, the present study has several limitations. First, the data were obtained from public databases, so the quality of the raw data cannot be appraised. Second, the sample size was relatively small, and the study failed to cover patients from different ethnicities and regions. In addition, the retrospective design may have caused inevitable inherent bias. Therefore, further studies with larger sample size and a prospective design are warranted to increase the statistical power and achieve more meaningful outcomes applicable to wider populations. Finally, although microarray-based bioinformatic analysis is a powerful tool to understand the molecular mechanisms and identify potential biomarkers, further experimental evidence is required to fully elucidate the underlying mechanisms related to HSPB8 expression in BC.

## Conclusion

In summary, the current study suggests that HSPB8 may be a promising diagnostic and prognostic molecular marker for BC. However, extensive prospective studies are required to verify the clinical application of HSPB8 in the personalized management of BC. Thus, further experimental validation should be performed to validate the biological role of HSPB8 in BC.

## Data Availability

The data used and analyzed during the current study are available from The Cancer Genome Atlas (TCGA) (https://portal.gdc.cancer.gov/) and Gene Expression Omnibus (GEO) (https://www.ncbi.nlm.nih.gov/geo). The names of the repository/repositories and accession number(s) can be found in the article/Supplementary Material.
